# Minimally Invasive and Proactive Approaches for Treatment of Acute Traumatic Brain Injury in Elderly Patients

**DOI:** 10.3390/jcm14145028

**Published:** 2025-07-16

**Authors:** Eiichi Suehiro, Tatsuya Tanaka, Akira Matsuno

**Affiliations:** Department of Neurosurgery, School of Medicine, International University of Health and Welfare, 852 Hatakeda, Narita 2868520, Chiba, Japan; s96047@hotmail.com (T.T.); akira.dr.ruby@gmail.com (A.M.)

**Keywords:** traumatic brain injury, elderly people, antithrombotic drugs, reversal therapy, blood biomarker, neuroendoscopy, craniotomy

## Abstract

The elderly population in Japan was 29.3% in 2024, the highest in the world, making medical care for elderly patients an urgent social issue. There are challenges in providing care for elderly patients with head injury, since the buffering effect of the expansion of the subdural space due to brain atrophy masks the neurological symptoms caused by a hematoma, making detection difficult. However, brain damage can be detected with high sensitivity and specificity using blood D-dimer as a biomarker without the need for head computed tomography (CT). Also, about 30% of elderly patients with traumatic brain injury (TBI) are taking antithrombotic drugs, and the effects of these drugs on TBI may include an increase in intracranial hematomas and an increased risk of deterioration. Reversal therapy is used as a countermeasure to prevent hematoma expansion, but this requires the administration of a reversal agent early after injury and before hematoma expansion. In decompression surgery, the use of a mini-craniotomy with neuroendoscopic assistance under local anesthesia can reduce invasiveness, and this method significantly reduces intraoperative bleeding and operation times compared to a major craniotomy. These innovations have improved mortality for TBI in elderly patients, but there is still a need for improvements in functional outcomes.

## 1. Introduction

The world population is aging rapidly. The proportion of people aged 65 or older in the total population (the aging rate) has risen from 5.1% in 1950 to 9.4% in 2020, and it is expected to rise to 18.7% in 2060, with aging expected to progress further over the next 40 years [1]. This aging trend has been considered a phenomenon in developed countries, but a similar situation is expected in developing countries in the future. Comparing the speed of aging based on the number of years required for the aging rate to reach 14% from 7%, it takes 115 years in France, 85 years in Sweden, 72 years in the United States, 46 years in the United Kingdom, and 40 years in Germany, while in Asian countries, it takes 24 years in Japan, 18 years in South Korea, and 15 years in Singapore [1]. The aging rate in Japan was 29.3% in 2024, the highest worldwide [1]. It is estimated that the aging rate in Japan will reach 33.3% in 2037 and 38.7% in 2070 [1]. In this rapidly aging society, how to deal with elderly medical care is an urgent social issue [1]. Reflecting these social changes, traumatic brain injury (TBI) cases in Japan are also changing. Data from the Japan Neurotrauma Data Bank (JNTDB) suggest yearly increases in elderly patients with severe TBI, with the rate of 51.7% in the JNTDB Project 2015 (2015–2017) showing that more than half of the severe TBI cases in Japan comprise these patients [2]. Along with these changes, the main mechanism of injury has changed from traffic accidents to falls, and the main type of TBI has changed from diffuse brain injury to acute subdural hematoma. This presents a major challenge for medical professionals in providing medical care that responds to the needs of an aging society. Japanese neurosurgeons should be in a position to report on our experiences and set an example for the world.

The outcome of TBI in elderly patients is worse than that in younger patients [3,4]. Factors related to the prognosis include age and impaired consciousness at the time of injury [5,6]. Therefore, in severe TBI in elderly patients, there is a tendency to assume a poor prognosis before treatment and to avoid aggressive treatment. However, the recent increase in elderly patients with TBI in Japan has led to more aggressive treatment being performed in these patients [7,8,9]. JNTDB data suggest that aggressive neurocritical care, including sedation, analgesia, airway management, intracranial pressure monitoring, temperature control, etc., has significantly reduced mortality from 62.7% to 51.1% in elderly patients with TBI [7]. Aggressive burr hole surgery and craniotomy have also improved the favorable outcome rate from 7% to 18% and mortality from 81% to 62% in these patients [9]. The outcome of TBI in elderly patients also varies depending on frailty before injury [10,11]. Thus, although the outcome in elderly patients is generally poor, favorable outcomes are possible with appropriate treatment and consideration of the condition before injury [12]. There is no need to avoid treatment because the patient is elderly, and appropriate treatment should be given after a sufficient discussion with the patient’s family [12]. In this article, we review treatment for TBI in elderly patients in the context of medical care in the super-aging society in Japan.

## 2. Pathogenesis and Clinical Characteristics of Elderly Patients with TBI

Falling is the most common mechanism of TBI in elderly people [13]. Elderly people are not very active outdoors, and falls often occur at home. The causes of falls are related to declines in physical functions such as sight, balance, and muscle strength; cognitive impairment; and hemiplegia due to stroke [14]. Drugs that increase the risk of falls include hypnotics, antidepressants, anxiolytics, opioids, and antipsychotics [14], as well as vasodilators, diuretics, beta blockers, alpha-adrenoreceptor antagonists, and dopaminergic agents that can cause orthostatic intolerance [15]. Thus, caution is required when using these medications. Morphologically, space-occupying lesions such as subdural hematoma and cerebral contusion are often seen in elderly patients with TBI [13]. These occur because the volume of the subdural space increases due to brain atrophy in elderly people, and the mobility of the cerebral hemispheres increases [14]. The movement of the cerebral hemispheres due to mild TBI damages bridging veins, resulting in subdural hematomas [14].

### 2.1. The “Talk and Deteriorate” Effect in Elderly Patients with TBI

Delayed deterioration is a characteristic of elderly patients with TBI [14]. The so-called “talk and deteriorate” effect is defined as a state in which a patient shows a relatively good neurological condition for a certain period after TBI, but then, it deteriorates to a severe neurological condition. The “talk and deteriorate” rate in elderly patients with TBI in the JNTDB is 21.4%, more than twice that of younger patients [13]. In elderly patients, thin hematomas are masked by the buffering effect of the expansion of the subdural space [14]. Also, the compliance of intracranial tissues is reduced, and this causes the impairment of consciousness to progress rapidly when hematoma expansion exceeds the intracranial buffering capacity [14], leading to a higher “talk and deteriorate” rate in elderly patients.

### 2.2. Difficulties in Initial Treatment of Head Injuries in Elderly Patients

The masking of a space-occupying lesion by the expansion of the subdural space causes difficulty with the initial treatment of elderly patients with TBI. For example, in patients aged 60 years or older, abnormal findings are found in head computed tomography (CT) in 17–57% of cases, even though the patients are conscious and exhibit clarity [16]. Elderly people often have a good level of consciousness, even if brain damage is shown on imaging [17]. Thus, it is difficult to identify brain damage based on the level of consciousness in the initial treatment of elderly patients with TBI. A head CT scan is needed to identify structural abnormalities, even if consciousness is good. The evaluation of the level of consciousness alone has low reliability in the diagnosis of brain damage in these patients, and new diagnostic tools are needed, in addition to neurological evaluations. Examples include the observation of abnormal behavior [18] and the measurement of blood biomarkers [19].

### 2.3. Checking Medications Taken During the Initial Medical Examination

In the initial treatment of elderly patients with TBI, it is important to check whether the patient is taking antithrombotic drugs. About 30% of such patients are thought to be taking these drugs [13], but there are cases in which the patient does not remember the details of their medications or cannot answer questions due to impaired consciousness. In Japan, patients are encouraged to bring along a notebook listing their medications. The “talk and deteriorate” rate in elderly patients with TBI increases from 21.4% to 30% with the use of antithrombotic drugs [13], and thus, the response to these drugs should be included in the treatment strategy.

## 3. Care for Elderly Patients with TBI Taking Antithrombotic Drugs

Since antithrombotic drugs are often taken by elderly patients with TBI, it is important to understand the effects of these drugs. Several observational studies in patients with severe TBI taking antithrombotic drugs have shown an increased incidence of fall as a mechanism of injury and a higher proportion of space-occupying lesions as a disease type [13,20]. Furthermore, antithrombotic drugs increase the “talk and deteriorate” rate and worsen the outcome [13,20]. Traumatic intracranial hemorrhage is more likely to occur in low-energy trauma such as falls due to the bleeding tendency caused by antithrombotic drugs, and the condition worsens due to the enlargement of the hematoma. These drugs have also been shown to be a significant risk factor for a “talk and deteriorate” status in elderly patients with TBI [21].

### 3.1. Reversal Therapy for Antithrombotic Drugs

It is important to manage antithrombotic drug use in the treatment of TBI in elderly patients, and this may include discontinuing antithrombotic drugs and reversal therapy [22]. Specific reversal agents, including vitamin K, fresh frozen plasma, four-factor prothrombin complex concentrates (4PCCs), idarucizumab, and andexanet alfa, can be used for each anticoagulant (Table 1). However, the combination of anticoagulant and reversal drug may differ depending on the circumstances of the medical insurance system of each country. Pharmacologically, these agents have a strong hemostatic effect [23,24] that leads to reductions in surgery time, length of stay in the intensive care unit, and in-hospital mortality in craniotomy [25,26]. However, there are no reports showing improved outcomes with anticoagulant reversal therapy [27]. Platelet transfusion and desmopressin are used for the reversal of antiplatelet drugs, but the effects of this reversal in patients with traumatic hematoma remain controversial [22]. A recent study found no differences in hemorrhage progression or the rate of neurosurgical intervention in patients with traumatic intracranial hemorrhage treated with and without platelet transfusion [28]. Platelet function tests are now recommended for identifying the benefits of antiplatelet agent reversal therapy and reducing the risk of thrombosis due to unnecessary treatment [29,30].

### 3.2. The Real World of Antithrombotic Reversal Therapy

We conducted an observational study on the current status of reversal therapy for elderly patients with TBI in a super-aging society [31]. The results showed that patients who received reversal therapy had significantly more severe TBI, a higher frequency of space-occupying lesions, and more frequent craniotomies and were more likely to be “talk and deteriorate” cases with poor outcomes [31]. These results were exactly the opposite of what we expected. A similar report from the United States also found that patients who received reversal therapy had more severe TBIs, more frequent craniotomies, and poorer outcomes [32]. These results suggest that reversal agents are being administered to severely ill patients after their condition has worsened, the hematoma has expanded, and craniotomy has become necessary.

### 3.3. Timing of Administration of Antithrombotic Reversal Agents

Neurons do not have the ability to regenerate, so it is important to intervene early before the condition worsens. Thus, early craniotomy for traumatic intracranial hemorrhage leads to a reduction in mortality [33], and the CRASH-3 study [34], which examined the hemostatic effect of tranexamic acid in TBI, also showed the effectiveness of early intervention in mild and moderate cases. In our observational study, the time from injury to the administration of the reversal agent was significantly shorter in patients with a favorable outcome (261.9 min) compared to those with a poor outcome (543.4 min) [31]. A correlation of outcome with time to the administration of the reversal agent has also been found in anticoagulant-related cerebral hemorrhage [35], with significantly decreased mortality and discharge to hospices for patients with a door-to-treatment time of ≤60 min [35]. Thus, earlier use of an antithrombotic reversal agent, before the condition becomes severe, is likely to prevent the worsening of TBI and lead to improved outcomes [31,36].

### 3.4. Ischemic Complications and Resumption of Antithrombotic Drugs

Ischemic complications are a concern in the administration of reversal agents in elderly patients with TBI who are taking antithrombotic drugs. The rate of ischemic complications due to reversal agents in these patients is 7.5% to 13.0% [25,31,36], and more than half of the patients had these complications develop more than one week after reversal therapy [31,36]. Thus, these cases probably include those with direct ischemic complications due to reversal agents, as well as those in which ischemic diseases developed due to the discontinuation of antithrombotic drugs. To avoid such complications, the early resumption of antithrombotic drugs may be beneficial. Guidelines recommend resuming anticoagulants within 2 weeks for high-risk cases and 7–8 weeks for low-risk cases [37]. However, earlier resumption may be possible, especially in cases of mild TBI in which the condition is stable. The timing of ischemic complications resulting from the discontinuation of antiplatelet drugs is often earlier than that of anticoagulants. In addition, most intracranial bleeding after trauma occurs within 3 days, so it is recommended that antiplatelet drugs be restarted 4 days after injury [38]. Thus, in elderly patients with TBI who are taking antithrombotic drugs, the early use of reversal agents and the early resumption of antithrombotic drugs are key to improving outcomes.

### 3.5. Indication Criteria for Reversal Therapy

Although it is desirable to administer a reversal drug while the condition is mild, this is difficult in many patients due to ischemic complications and medical costs. Thus, there is a need to identify cases of mild TBI in which the condition is expected to worsen. Contrast CT images of extravasation have been suggested to be predictive of hematoma growth and poor prognosis [39,40], and we have shown that prognosis prediction is possible using the blood D-dimer level as a biomarker, which is inexpensive and can be used anywhere [41]. However, this level is affected by anticoagulants, medical history, and trauma to other sites, so its use is limited. This problem may be solved through the prognostic prediction of “talk and deteriorate” cases using blood biomarkers specific to nerve damage, such as glial fibrillary acidic protein (GFAP) and ubiquitin C-terminal hydrolase L1 (UCH-L1) [42]. Finally, 15% of elderly patients with TBI who are taking antithrombotic drugs may not be receiving the appropriate drugs [43]. These patients are gaining no benefit from these drugs but still have an increased risk of cerebral hemorrhage due to TBI. Thus, there is a need to raise awareness on the use of appropriate antithrombotic drugs to improve the prognosis of elderly patients with TBI.

## 4. Blood Biomarkers as an Alternative Diagnostic Tool to Head CT

Japan has the highest number of CT scanners in the world, at about 100 per million people [44], and the threshold for taking a head CT scan is low. The amount of ionizing radiation from a head CT is 2.0 mSv (200 mrem), which is roughly the same amount that a person receives in one year from background radiation and 66% of the radiation exposure experienced by a flight crew member from air travel. The number of CT scans is increasing, and there are concerns that such low-dose radiation exposure may increase the risk of cancer. If current practices continue, CT-related cancers may eventually account for 5% of new cancer diagnoses per year [45]. In Japan, symptoms are unlikely to appear early after injury in elderly patients, so head CT scans are strongly recommended and are now the highest medical cost for elderly patients with TBI [46]. Furthermore, CT scans present problems, such as geographical issues and increased burden on medical staff. A simple diagnostic tool to replace head CT scans is needed, and this role may be fulfilled by the use of blood biomarkers. In situations where the patient’s condition requires urgent treatment, a head CT scan is the first choice, but in asymptomatic head trauma patients who are typically elderly, triage based on blood biomarkers may be appropriate.

### 4.1. Diagnosis of TBI Using Blood D-Dimer

We have reported the usefulness of blood D-dimer as a biomarker for the diagnosis of structural abnormalities in mild head injuries [47]. The measurement of blood D-dimer is routine in daily medical practice, and it is simple and inexpensive. Nerve damage caused by TBI releases nerve-derived tissue factors into blood, and the blood D-dimer level rises due to the activation of the extrinsic coagulation cascade [48]. Using a cutoff for blood D-dimer of 1.5 μg/mL, it is possible to identify structural abnormalities in the skull with a sensitivity of 77.4% and a specificity of 89.5%, making this a useful diagnostic aid in TBI [47]. We also showed that it is possible to predict “talk and deteriorate” cases using blood D-dimer values [41], and based on these results, we created a post-hospitalization treatment protocol for patients with mild to moderate TBI using the blood D-dimer level at the time of admission [41]. However, D-dimer levels can also increase when there is trauma to the trunk or limbs, myocardial infarction, disseminated intravascular coagulation, deep vein thrombosis, and pregnancy, and it is necessary to exclude these conditions in the evaluation of patients with TBI.

### 4.2. TBI Diagnosis Using Neurospecific Biomarkers

Neuron-derived biomarkers such as GFAP, UCH-L1, and S-100β have attracted recent attention in TBI treatment [19]. These markers may predict “talk and deteriorate” cases [42], but their use in elderly patients with TBI is difficult because GFAP and UCH-L1 are generally higher in elderly patients, which reduces diagnostic accuracy [49,50]. Changes in these markers may also be small in the acute phase of head injury [51], and further data are needed in elderly patients.

## 5. Minimally Invasive Treatment Strategies for Elderly Patients with TBI

The purpose of craniotomy for acute subdural hematoma is to reduce intracranial pressure (ICP) and ensure hemostasis. The bleeding sources of acute subdural hematoma are diverse, including the parietal bridging vein, the vein of Labbe, and the Sylvian vein. To ensure hemostasis, it is important to expose these bleeding sources, and in principle, this requires a large craniotomy.

### 5.1. Usefulness of Burr Hole Surgery for Acute Subdural Hematoma

In acute subdural hematoma, the stress of surgery, including excessive intraoperative bleeding, long operation time, and general anesthesia, is a significant factor in worsening the outcome [52]. In particular, highly invasive treatments such as large craniotomy place a heavy burden on elderly patients and have a significant impact on outcomes. In contrast, burr hole surgery is a safe, effective, and simple life-saving procedure for treating acute subdural hematoma that is used in resource-limited areas [53]. We examined the effectiveness of burr hole surgery from the perspective of decompression by placing an ICP sensor before burr hole surgery and examining ICP after surgery [54]. In all seven cases examined, ICP decreased to the normal range after surgery [54]. Although burr hole surgery alone cannot fully remove subdural hematoma, these findings show that ICP can be rapidly reduced by simply aspirating and removing the liquid components of the hematoma and cerebrospinal fluid. By taking advantage of the period of ICP reduction, coagulation disorders and the general condition can be improved. With this one step, the amount of bleeding and required blood transfusion during surgery and the burden on the patient are reduced [54].

### 5.2. Mini-Craniotomy with Neuroendoscopy

The number of cases of mini-craniotomy for acute subdural hematoma in elderly patients is increasing. Mini-craniotomy has a lower hematoma removal rate than major craniotomy, but the operation time is significantly shorter, and mini-craniotomy is not inferior to large craniotomy in terms of functional outcome [55]. However, there is a concern that the bleeding site cannot be exposed during mini-craniotomy, which may lead to difficulty in hemostasis. Neuroendoscopes are used to compensate for the lower hematoma removal rate or difficulty in hemostasis due to mini-craniotomy [56]. The use of mini-craniotomy under local anesthesia and the final checking of the subdural space with an endoscope permits sufficient hematoma removal, the confirmation of hemostasis at the bleeding source, and the avoidance of complications related to intubation and artificial respiratory support [56]. In addition, surgery performed under local anesthesia allows patients to wake up well after surgery and to leave the bed the day after surgery, resulting in a good rehabilitation effect. By performing a mini-craniotomy centered on the contrast-enhanced area in a preoperative contrast CT scan, it is possible to confirm the bleeding source, and safe hemostasis can be achieved under direct vision [57]. These benefits are likely to be of value in elderly patients with TBI, for whom minimally invasive treatment is preferable.

In Japan, such methods have been used to treat elderly patients with TBI. Mini-craniotomy has been shown to reduce the surgical time and intraoperative bleeding compared to large craniotomy but with no improvements in functional outcomes [58]. Although the burden on elderly patients is reduced by this method, the lack of standardized treatment eligibility criteria may account for the observed failure to improve functional outcomes [59]. At present, this method is used for a variety of pathological conditions with different severities, and this may explain why functional outcomes are inconsistent. Yokosuka et al. [60] have proposed an indication of symptomatic patients aged ≥70 years without cerebral contusion and without progressive hematoma expansion or a bleeding tendency. The degree of brain atrophy also seems to be an important factor in the suitability of this surgical method [61]. Miki et al. [62] reported that only a few cases among many neuroendoscopic surgeries required reoperation due to rebleeding or the expansion of hematoma. Risk factors for rebleeding and hematoma enlargement with this method include early surgery within 6 h after injury (incomplete hemostasis), cases with cerebral edema accompanied by a midline shift greater than the thickness of the hematoma, and findings of skull fracture or traumatic subarachnoid hemorrhage [62]. There is a need to establish surgical indications that include these conditions and a unified severity level and to examine outcomes under these conditions.

## 6. Future Directions

In the future, population aging will spread throughout the world, including emerging countries. This is not a problem that only concerns others but one that we must address as a problem that applies to our own country as well. Japan would like to be a role model for this.

First of all, we must recognize that the pathology of TBI in elderly people is different from that of TBI in young people. In other words, we need to treat them differently than before. In this context, the evaluation of TBI patients is important. Until now, the severity of injury was determined only using the Glasgow Coma Scale (GCS) score. However, in recent years, patients have been evaluated from a more multifaceted perspective. This is because, especially in elderly patients, the level of consciousness during initial treatment does not accurately reflect the severity of injury. The CBI-M framework is used as a new characterization that evaluates not only the GCS score but also pupil findings, blood biomarkers, the findings of images, and so on from multiple angles [63]. In addition, it will be necessary to evaluate dementia and frailty, factors specific to the elderly. Using such new evaluations, the pathology of TBI in elderly people should be evaluated earlier, and early intervention should be performed in cases where it is necessary. Furthermore, we described antithrombotic measures as a characteristic of initial medical care for elderly patients with TBI. First, it is necessary to determine whether or not the patient is taking antithrombotic drugs and what type of drugs they are taking. Because the patient is often unconscious or disturbed, they are often unable to explain what medications they are taking. In the future, we await the development of a method for predicting what medications are being taken with a simple test [64]. In addition, there is a need to provide clear evidence regarding the effectiveness and usage of antithrombotic reversal drugs.

To improve the outcome of elderly patients with TBI, a treatment specific to the elderly, that is, a non-invasive treatment, is necessary. In Japan, surgery using mini-craniotomy with neuroendoscopy is preferred, but unfortunately, its effectiveness in improving outcomes has not been proven. It is minimally invasive and has a short operation time, and the hematoma removal rate is comparable to that of large craniotomies [56,58]. In the future, clinical research should be conducted to clearly define the surgical indications for mini-craniotomies and demonstrate their effectiveness.

In recent years, cell therapy for TBI has been reported to improve motor function [65], and post-rescue treatment is also expected to improve functional prognosis. These functional recovery methods do not rely solely on cell therapy, but they are effective when combined with rehabilitation. In the future, it is expected that functional recovery treatments will be established in the chronic phase of TBI.

This review summarizes the challenges of treating elderly patients with TBI based on the experience of Japan, which is an ultra-aging society. Therefore, this study has limitations in that our situation is not the same as those around the world. For example, it would be difficult to take head CT scans as frequently as in Japan in many countries. In addition, there are differences in the drugs that can be used for antithrombotic reversal therapy due to differences in insurance medical care systems. Regarding the use of neuroendoscopes, the type of equipment varies depending on the region. However, by disseminating evidence of TBI treatments for elderly people in the future, we hope to make these treatments the global standard of care, leading to improved prognosis for elderly patients with TBI.

## 7. Conclusions

The key strategies for elderly patients with TBI are early diagnosis and early intervention, followed by reducing the burden on patients through minimally invasive surgery.

In elderly patients with TBI, brain damage may be observed even if there is no impaired consciousness. Whenever possible, a head CT scan should be taken to check for intracranial damage. However, excessive head CT scans can cause various problems. To avoid these, in elderly patients with TBI who do not have impaired consciousness, triage can be performed using blood biomarkers. Elderly people have a high rate of taking antithrombotic drugs. Head trauma patients who take antithrombotic drugs have a high rate of “talk and deteriorate” and poor outcomes. Antithrombotic drug reversals should be administered as early as possible. In addition, non-invasive methods should be selected for surgical procedures to reduce the burden on patients. We perform small craniotomy surgery under neuroendoscopy.

These strategies have improved outcomes, since the mortality of elderly patients with TBI has decreased significantly from 62.8% in Project 1998 to 44.7% in Project 2015 [8]. However, the proportion of dependent survivors has increased from 23.2% in Project 1998 to 39.1% in Project 2015 [8], which suggests a need to improve the functional prognosis, in addition to mortality. A thorough discussion with the family of the patient, including an evaluation of the activities of daily living before the injury, and the provision of aggressive and appropriate treatment are both important for elderly patients with TBI.

## Figures and Tables

**Table 1:** Antithrombotic drugs and their reversal agents.

Antithrombotic Drugs	Reversal Agents
Warfarin	Vitamin K Fresh frozen plasma Prothrombin complex concentrate
Heparin/LMWH	Protamine sulfate
Direct thrombin inhibitor	Idarucizumab Activated charcoal (within 2 h after taking) Hemodialysis
Factor Xa inhibitor	Activated charcoal (within 2 h after taking) Andexanet alpha
Antiplatelet drugs	Platelet transfusion (if surgical procedure is required)

## Abbreviations

The following abbreviations are used in this manuscript:

CT	Computed tomography;
TBI	Traumatic brain injury;
JNTDB	Japan Neurotrauma Data Bank;
GFAP	Glial fibrillary acidic protein;
UCH-L1	Ubiquitin C-terminal hydrolase L1;
ICP	Intracranial pressure;
GCS	Glasgow Coma Scale.
